# WHEN STRANGERS BECOME FAMILY: THE ROLE OF CIVIL SOCIETY IN ADDRESSING THE NEEDS OF AGING POPULATION

**DOI:** 10.1093/geroni/igz038.252

**Published:** 2019-11-08

**Authors:** Ronald J Angel

**Affiliations:** University of Texas, Austin, Austin, Texas, United States

## Abstract

Mexico’s rapidly aging population presents serious short and long-term challenges to the state and to families, since relatively few individuals have formal retirement plans. Although health care access is formally universal, and non-contributory retirement income is provided to all elderly in need, the Mexican old-age welfare remains limited. In this study we assess the potential role of civil society organizations (CSOs), a category that includes secular non-governmental organizations (NGOs), faith-based organizations (FBOs), in advocacy for and service provision to elders in need. Social and demographic changes, including the migration of children away from their parent’s community, the need for women to work, smaller families, and more are undermining the capacity of the family to provide all of the care and support that frail aging parents need. Given the fact that the federal, state, and municipal governments are limited in what they can provide, the role of CSOs is potentially significant.

